# The Association Between Family Function and Adolescents' Depressive Symptoms in China: A Longitudinal Cross-Lagged Analysis

**DOI:** 10.3389/fpsyt.2021.744976

**Published:** 2021-12-17

**Authors:** Enna Wang, Junjie Zhang, Siya Peng, Biao Zeng

**Affiliations:** ^1^School of Education, Tianjin University, Tianjin, China; ^2^Collaborative Innovation Center of Assessment for Basic Education Quality, Beijing Normal University, Beijing, China; ^3^College of Psychology, Shenzhen University, Shenzhen, China; ^4^State Key Laboratory of Cognitive Neuroscience and Learning, International Data Group (IDG)/McGovern Institute for Brain Research, Beijing Normal University, Beijing, China

**Keywords:** adolescents, family function, depressive symptom, cross-lagged analysis, circular effect

## Abstract

The complex interrelationships between family function and adolescents' depressive symptoms are not yet fully clarified, especially in China. Based on the family systems theory, this study explored the relationships between family function and Chinese adolescents' depressive symptoms by a 3-year longitudinal study design. Three waves of data were collected from 1,301 Chinese middle school students in Grade 7 to Grade 9. All participants completed the Chinese Family Assessment Instrument (CFAI) and the Center for Epidemiologic Studies Depression Scale (CES-D) once a year during the junior middle school period. Our results showed that both family function and adolescent depressive symptoms were stable in Grade 7 and Grade 8, but in Grade 9, family function increased and depressive symptoms declined. Furthermore, we found that the family function in Grade 7 negatively influenced depressive symptoms of adolescents in Grade 8, while adolescent depressive symptoms in Grade 8 negatively impacted subsequent family function in Grade 9, namely there was a circular effect between family function and adolescent depressive symptoms. These findings suggest that the associations between family function and adolescents' depressive symptoms are dynamic and time-dependent. Our study contributes to the intervention aimed at the reduction of adolescent depressive symptoms from the family perspective.

## Introduction

Depression has become an alarming health issue among adolescents, with typical symptoms such as feelings of sadness, decreased interest, and suicidal thoughts (1, 2). In China, previous reports from the National Health Commission (3) pointed that, ~30 million teenagers exhibited varied degrees of emotional disorders, with depressive symptoms the most common. A recent study showed that the prevalence rates of depressive symptoms among early adolescents are rising rapidly, up to an astonishing incidence rate of 24.3% (4). As a prevalent psychological disorder, adolescent depression was significantly associated with a series of impairments in cognitive, psychological and social functioning, such as academic failure (5), interpersonal problems (6), and even self-injury and suicide (7, 8). Evidence from the Scar Hypothesis of depression had also assumed that depressive symptoms had a long-lasting deleterious effect on adolescents' self-concept and personality, leaving a “scar” on an individual's self-esteem (9–11). Furthermore, going through depression during early adolescence could even result in an increased risk of other health issues in adulthood (12–14).

Given these negative effects of adolescent depression, substantial researches have addressed which factors are implicated in the deterioration of depressive symptoms. Existing evidence suggests that both individual and environmental characteristics have made their contributions to adolescents' depressive symptoms (15, 16). Among these factors, the family function has drawn substantial attention due to its vitally important role in adolescent development (17–19). However, previous studies focused more on the unidirectional effect of family function on adolescents' depressive symptoms (16, 20), giving less consideration to the potential negative effect of adolescent depressive symptoms on the whole family system and the potential bidirectional relations between them. Additionally, the majority of them adopted cross-sectional data (21, 22), or short-term longitudinal data (23), from which the dynamic interaction between family functioning and adolescent depressive symptoms could not be well-demonstrated. Therefore, it is particularly necessary to conduct a longitudinal study to clarify the possible dynamic association between family function and adolescent depressive symptoms, especially in the Chinese context, where people take the family of great importance.

The family function generally indicates the quality of family life involving a family's competence, wellness, as well as strengths and weaknesses (24). Taking the family system as a whole, this concept is above and beyond both the dual parent-child relationship and the binary conjugal relationship (25). Family function encompasses three core dimensions: mutuality, communication, and harmony (26), which have been extensively employed in the related studies of the family domain (27, 28). A large body of studies has revealed that family function plays a critical role in an individual's healthy development. For example, one prospective study showed that harmonious family relationships and good parent-child communication could significantly promote positive developmental attributes (28), which is expected to be beneficial for the academic achievement of adolescents (29). On the contrary, teenagers who live in an impaired family are more likely to experience internalizing problems such as depression, anxiety, and withdrawal (30), and show externalizing problems such as antisocial, aggressive and disobedient behaviors (31).

To date, numerous studies have been conducted to investigate the relationship between family function and adolescent depressive symptoms, in which scholars found that these two factors were negatively correlated (32, 33). As for the direction of the association between them, the majority of extant studies supported the family-driven effect (family function influences adolescent depressive symptoms), in which researchers regard family function as an important predictor of adolescent depressive symptoms. For example, some scholars found that adolescents living in highly dysfunctional families were prone to have negative self-cognition, which was a key trigger to the emergence of depressive symptoms in adolescents (34, 35). Similar results were found among teenagers in China (36, 37). A recent Chinese study, using a sample of 11,865 adolescents, has also found that impaired family function might increase the risk of adolescent internalizing problems like depression, and researchers further point out that the influence of family function on adolescent depression was partly mediated by positive youth development attributes (28). With the awareness of the dynamic development of adolescent depressive symptoms, some scholars began to pay attention to the longitudinal influence of family factors (e.g., family relationships, family social support, and family functioning) on depressive symptoms (38, 39). For instance, one current research found that poor family functioning at baseline could significantly predict depressive symptoms of junior high school students 1 year later (16). Nonetheless, the above studies have the common point of treating family function as a static antecedent variable, failing to address the potential reverse influence from the adolescent depressive symptoms to the whole family.

According to the family systems theory (40, 41), the plight of the family systems could induce the occurrence of individual psychological problems, individual mental health problems might also put the family at risk, resulting in poorer communication, worse cohesion, and more conflicts and arguments (23). Thus, apart from the family-driven effect, there might be still two possible effects: the child-driven effect (adolescent depressive symptoms influence family function), and the potential reciprocal effect (reciprocal influence of family function and adolescent depressive symptoms). Although early studies have demonstrated the destructive effects of individual mental distress on the family, most of them used clinical samples. For example, a study using 424 depressed patients indicated that more severe depressive symptoms were positively related to subsequent more family arguments for both men and women (42). In fact, for depressed adolescents, their emotional problems might also damage the family function. The “below” pressure hypothesis also provides some insight into this child-driven effect (43). It assumes that adolescent maladjustment such as depressive symptoms could serve as the “below” pressure, putting a huge strain on their parents. Thus, family conflict might be introduced (44), and the negative emotions would further spread through the whole family (45), hindering the family system from functioning well. However, as far, empirical researches examining the child-driven effect among the subclinical adolescent samples were relatively rare. From childhood to adolescence, early adolescents experienced great change on cognitive, emotional, and social levels. During this special turning period, the development risk of internalization or externalization of adolescents greatly increased (46, 47), which will undoubtedly put the whole family under pressure (48, 49). Especially when adolescents experienced emotional problems, the function and satisfaction of the family were threatened (23, 50).

Theoretically, the relationship of family factors and individual emotional distress was likely bidirectional in nature. In fact, a handful of studies have supported the reciprocal effect. For example, in a 3-year longitudinal study that involved 451 early adolescents and their families, researchers found a reciprocally interrelated association of marital conflict and adolescent depressive symptoms (51). Likewise, other scholars also found a significant cross-lagged relationship between parent-child hostility and adolescent depression for mother and daughter (52). However, those studies were mainly conducted in particular dyadic or family subsystems. Empirical evidence on the reciprocal linkage between family function (on the whole family system level) and adolescent depressive symptoms is insufficient and inconsistent. Based on both Victoria and Washington samples in America, Kelly et al. (50) found that family conflict and adolescent depressed mood were bidirectionally linked over time, which was independent of the factors outside the family, such as school bullying or academic performance. Whereas, in a more recent study, Mastrotheodoros et al. (23) fail to certify the reciprocal model, with results in the cross-lagged panel models showing a unidirectional association from internalized problem to family functioning at the 6-month time interval. Since these studies were carried out based on the context of Western culture, we still know little about the cause or effects of family function on Chinese teenagers' depressive symptoms. As a country emphasizing collectivism, Chinese youth are deeply influenced by family (53). The role of the family in the development of adolescents is particularly prominent, and different from that of western culture in many respects (54, 55). Therefore, it is certainly worthy to reveal the longitudinal relationship between family function and Chinese adolescent depressive symptoms in a relatively long period of time.

Guided by the family system theory, the present study adopts a 3-year longitudinal tracking design, attempting to investigate the possible longitudinal associations between family function and adolescent depressive symptoms in the context of Chinese culture. The current study is anticipated to offer some implications for the intervention to interrupt the progression of adolescents' depressive symptoms, as well as the improvement of their family function. The hypotheses of this study are as follows: (i) family function and depressive symptoms would change during the three junior high school years; (ii) family function significantly affect adolescent depressive symptoms; (iii) adolescent depressive symptoms significantly influence family function; (iv) family function and adolescent depressive symptoms might have a reciprocal relationship.

## Materials and Methods

### Participants

Data for the current study were based on three measurement waves, which were collected in October 2016 (T1; when adolescents just entered into junior school), 1 year later (T2; when adolescents had spent 1 year in junior school) and 2 years later (T3; when adolescents were in junior grade three). We conducted surveys in five middle schools from nine districts in Shenzhen, Guangdong Province, China by random selection. At the first wave, 1,544 adolescents with a mean age of 12.46 years old (SD = 0.63) participated in the initial study. At the subsequent waves, the sample sizes of participates were 1,511 for T2, and 1,480 for T3, respectively. Non-response at T2 and T3 was mainly for the reason that these students were absent during the survey days or they had moved to other schools. Therefore, our final analytical sample included a total of 1,301 students who completed all the items of two main study variables at three measurement waves.

### Procedure

Adolescents were invited to attend a paper-and-pencil test in classroom settings during regular school hours. Before each measurement, research assistants introduced the aim of the study, the procedures of the test, as well as the confidentiality safeguards. After receiving this information, students decided to participate in or withdraw from the study. If they agreed upon participation, adolescents and their parents should provide informed consent. At all measurements, written informed consent was obtained. The research assistants were present during the 20-min test to supervise the whole data collection, and answer students' questions about the test at every wave of surveys. No compensation was offered to the participants. Both the Human Research Ethics Committee of the affiliated institution and the administrative committees of the surveyed schools approved the questionnaires and procedures.

### Instruments

#### Chinese Family Assessment Instrument

The refined version of the Chinese Family Assessment Instrument was used to measure adolescents' family function (26). This scale contains 9 items, with each of the three items measuring mutuality, communication, harmony and conflicts, respectively. A sample item for mutuality sub-scale is “My family lives in harmony,” for communication sub-scale is “In general, parents and children often have conversations,” and for harmony and conflicts sub-scale is “We have a lot of friction.” Participants were instructed to rate each item from 1 (“very dissimilar”) to 5 (“very similar”). After reversing the conflict sub-scale scores, the means of the nine items were computed, with a higher score representing a healthier family function. In this study, the Cronbach's alpha coefficient of the CFAI was 0.86 at Time 1, 0.85 at Time 2, and 0.89 at Time 3.

#### Center for Epidemiologic Studies Depression Scale

Adolescent depressive symptoms were assessed using the Chinese version of the Center for Epidemiological Studies Depression Scale (56, 57). It consists of 20 items, with a Likert rating scale from 0 to 3, representing “rarely or none of the time,” “sometimes [1–2 days],” “often [3–4 days],” “most or all of the time [5–7 days],” respectively. An example is “I was bothered by things that usually don't bother me.” Participants make choices on each statement to assess the frequency they had experienced these depressive symptoms in the last week. Participants who scored higher in the CES-D were considered to have a higher level of depressive symptoms. In the current study, the Cronbach's alpha coefficients for the CES-D at Time 1, Time 2, and Time 3 were 0.85, 0.85, and 0.88, respectively.

### Demographic Variable

Adolescents' demographic information were measured by a questionnaire including the adolescents' gender (1 = boy and 2 = girl), age, place of birth (1 = rural area, 2 = Shenzhen, and 3 = other cities), only child or not (1 = only-child, 2 = non-only child), parents' education level (1 = middle school or below, 2 = high school or vocational college, 3 = university, and 4 = above university), and per capita monthly family income (CNY) (1 = <1,000, 2 = 1,000~1,999, 3 = 2,000~2,999, 4 = 3,000~3,999, 5 = 4,000~4,999, 6 = 5,000~5,999, 7 = 6,000 or above). Table 1 summarizes the demographic characteristics of participants.

**Table 1:** Demographic characteristics of participants.

	***n*** **(%)/** **mean (SD)**	
Age (years)	12.46	0.63
**Sex**		
Male	666	51.19%
Female	621	47.73%
Missing	14	1.08%
**Only child**		
Yes	499	38.36%
No	799	61.41%
Missing	3	0.23%
**Place of birth**		
City	1,141	87.70%
Rural	158	12.14%
Missing	2	0.15%
**Father's education level**		
Junior school and below	404	31.05%
Senior school	451	34.67%
Bachelor's degree	226	17.37%
Above a bachelor's degree	120	9.22%
Missing	100	7.69%
**Mother's education level**		
Junior school and below	492	37.82%
Senior school	427	32.82%
Bachelor's degree	217	16.68%
Above a bachelor's degree	74	5.69%
Missing	91	6.99%
**Per capita monthly family income (CNY)**		
<1,000	27	2.08%
1,000~1,999	81	6.23%
2,000~2,999	135	10.38%
3,000~3,999	169	12.09%
4,000~4,999	121	9.30%
5,000~5,999	127	9.76%
6,000 or above	439	33.74%
Missing	202	15.53%

### Data Analysis

As usual, we input all data into the SPSS25.0. Then, the means and standard deviations of the two main study variables were computed, and one-way repeated measures ANOVA was applied to examine the changes in family function and depressive symptoms across the three-time points. We also calculated correlations between family function and depressive symptoms. Next, since the current study adopted a repeated-measure design, measurement invariance testing was conducted to examine if the constructs of family function and depressive symptoms kept invariant over 3 years. After doing this, we performed an auto-regressive cross-lagged (ARCL) model to check the effects of family function and adolescent depressive symptoms in Mplus 8.3. ARCL analyses contained both cross-lagged effects and autoregressive effects. It allowed us to examine the potential influence of one construct (e.g., family function) on another (e.g., depressive symptoms) at a later time point (cross-lagged effects), controlling for the regression of both constructs on themselves assessed at the previous time points (auto-regressive effects). In ARCL analysis, demographic variables were taken as covariates. Due to the non-response on several demographic variables for some adolescents, the full information maximum likelihood (FILM) estimation for unbiased estimates was used to handle the missing demographic data (58).

To examine the goodness of model fit, a series of criteria were estimated, including chi-squared index, comparative fit index (CFI), Tucker-Lewis index (TLI), root mean square error of approximation (RMSEA) with 95% confidence intervals (95% CI), and standardized root mean square residual (SRMR). We considered the model fit acceptable if 1) normed chi-square (χ^2^/df) was <5; 2) the values of CFI and TLI were more than 0.90; 3) the values of RMSEA and SRMR were < 0.08 (59). Additionally, because that family function and depressive symptoms were self-reported by the adolescents themselves, there might exist common method biases in the present study. Thus, we conducted Harman's single-factor test to examine the possible common method variance (60).

## Results

### Testing of Common Method Variance

The Harman's single-factor analysis was for the test of the common method variance effect. The results showed that 6, 5, and 5 factors had eigenvalues >1 for the three time-point assessments, and that the rates of the first factors accounting for the amount of variation were all below 40% (25.36, 24.87, 32.46%, respectively). These results indicated that the common method biases could be ignored in this study.

### The Development Trend of Family Function and Adolescent Depressive Symptoms Over 3 Years

One-way repeated measures ANOVA was used for the difference of family function and adolescent depressive symptoms among the three measurement times, respectively (see Table 2). For family function, a significant time effect was observed, *F*_(1.93, 2510.36)_ = 14.47, *p* < 0.001. Pairwise comparisons indicated that the difference of family function scores was not significant between T1 and T2 (*p* > 0.05), but the scores of family function at T3 were significantly higher than T1 (*p* < 0.001) and T2 (*p* < 0.001). For adolescent depressive symptoms, a significant effect of time was observed, *F*_(1.94, 2525.51)_ = 12.53, *p* < 0.001. Further analyses showed that the difference of adolescent depressive symptoms scores was not significant between T1 and T2 (*p* > 0.05), while the scores of adolescent depressive symptoms at T3 were significantly lower than T1 (*p* < 0.001) and T2 (*p* < 0.001).

**Table 2:** Descriptive statistics for family function and depressive symptoms.

**Time**	**Family function**		**Depressive symptoms**	
	**M**	**SD**	**M**	**SD**
T1	4.07	0.77	13.66	9.15
T2	4.04	0.79	13.76	9.33
T3	4.17	0.77	12.40	9.31

*T1, Time 1 (first year); T2, Time 2 (second year); T3, Time 3 (third year)*.

### Analysis of Correlation Between Family Function and Adolescent Depressive Symptoms

Table 3 showed the correlation coefficient between family function and adolescent depressive symptoms in the three surveys. The results revealed that family function significantly negatively correlated with adolescent depressive symptoms both synchronously and longitudinally. The synchronous correlation coefficient ranged from −0.30 to −0.47, and the longitudinal correlation coefficient ranged from −0.13 to −0.30. The preliminary findings suggested that the adolescent depressive symptoms arise as the family function declined, and vice versa. Additionally, adolescent depressive symptoms were positively associated with each other during 3 years, with the correlation coefficient ranged from 0.25 and 0.42. Meanwhile, family functions were positively to each other over the 3 years, with the correlation coefficient ranged from 0.26 to 0.43.

**Table 3:** Correlations of family function with adolescent depressive symptoms.

**Variables**	**T1 FF**	**T2 FF**	**T3 FF**	**T1 Dep**	**T2 Dep**	**T3 Dep**
T1 FF	1					
T2 FF	0.26**	1				
T3 FF	0.43**	0.24**	1			
T1 Dep	−0.37**	−0.13**	−0.30**	1		
T2 Dep	−0.19**	−0.30**	−0.16**	0.25**	1	
T3 Dep	−0.29**	−0.13**	−0.47**	0.42**	0.25**	1

*T1, Time 1 (first year); T2, Time 2 (second year); T3, Time 3 (third year); FF, Family Function; Dep, Depressive symptoms*.

** *p < 0.01*.

### Measurement Invariance of Family Function and Depressive Symptoms

Measurement invariance of the two main study variables at three-time points was examined. The configural invariance model (M0), factor loading invariance model (M1) and residual invariance model (M2) of family function and depressive symptoms were established, respectively. For family function and depressive symptoms, the fitting indexes of each model were all met the criteria (see Table 4). On the advice of Cheung and Rensvold (61), compared with chi-square values, ΔCFI was a more stable indicator for model comparison since it is less influenced by model parameters and sample size. Therefore, this study used the Δ CFI index for model comparison. If Δ CFI is >0.01, we declined to the hypothesis of measurement invariance (61). In this study, model comparison results suggested that both family function and depressive symptoms showed measurement invariance across three-time points (Δ CFI < 0.01). Thus, the cross-lagged analysis could be carried out in the next step.

**Table 4:** Model fitting results of measurement invariance for family function and depressive symptoms.

	**χ^2^**	**df**	***p***	**CFI**	**TLI**	**RMSEA (90% CI)**	**SRMR**	**ΔCFI**
**FF**								
M0	36.388	15	<0.001	0.995	0.989	0.033 (0.019, 0.047)	0.027	
M1	65.291	19	<0.001	0.990	0.981	0.043 (0.032, 0.055)	0.046	0.005
M2	69.999	23	<0.001	0.990	0.984	0.040 (0.029, 0.050)	0.046	0.000
**Dep**								
M0	81.748	39	<0.001	0.993	0.988	0.029 (0.020, 0.038)	0.029	
M1	98.411	45	<0.001	0.991	0.987	0.030 (0.022, 0.038)	0.032	0.002
M2	153.583	51	<0.001	0.984	0.979	0.039 (0.032, 0.047)	0.034	0.007

*FF, Family Function; Dep, Depressive symptoms; M0, configural invariance model; M1, factor loading invariance model; M2, residual invariance model*.

### Autoregressive Cross-Lagged Analysis of Family Function and Adolescent Depressive Symptoms

**Figure 1:**
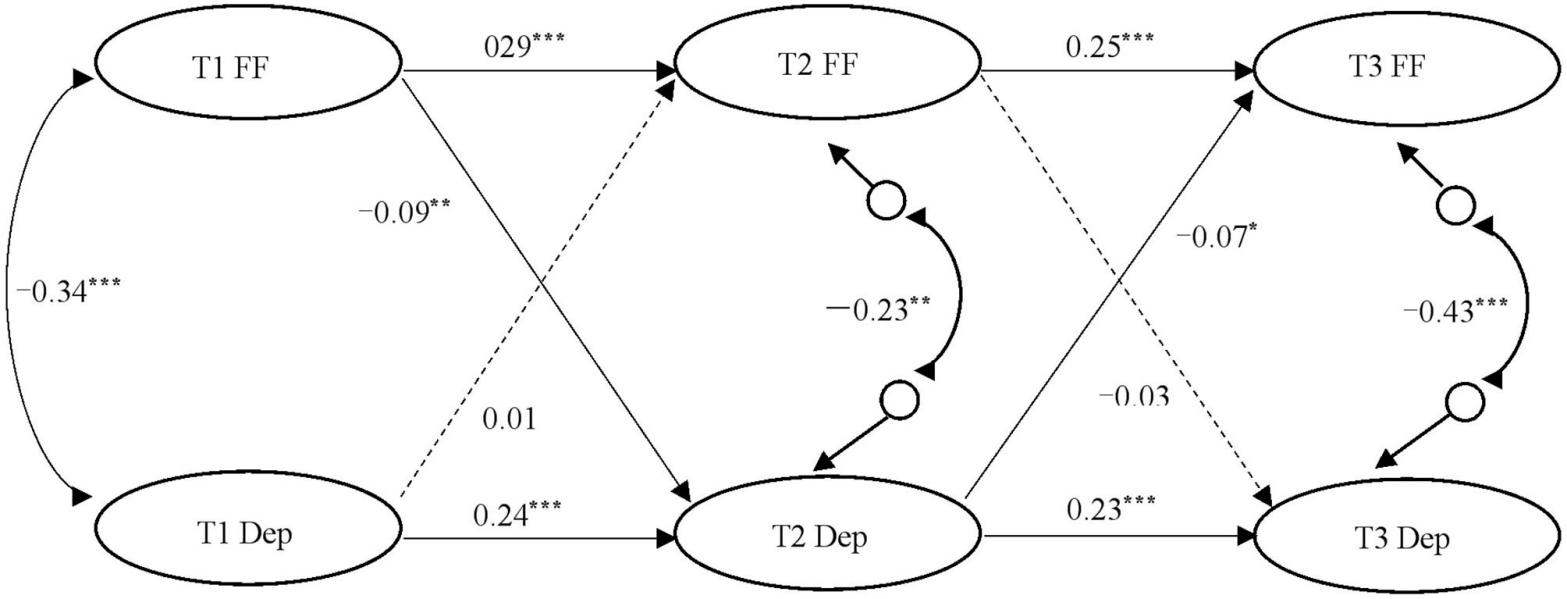
The ARCL model of family function and adolescent depressive symptoms. T1, Time 1 (first year); T2, Time 2 (second year); T3, Time 3 (third year); FF, Family Function; Dep, Depressive symptoms. Solid line = significant coefficient; Dotted line = non-significant coefficient. All coefficients were standardized. Control variable was not exhibited in the figure for the simplicity of the model. *p < 0.05; **p < 0.01; ***p < 0.001.

First, adopting the unique information method (62), we packaged family functions into three observed items (mutual relationship, communication and adaptation, conflict and harmony), and depressive symptoms into four observed items (depression, positive emotion, somatic symptoms, and interpersonal problems). Then, we employed structural equation modeling (SEM) with latent variables to analyze the cross-lagged relationship between family function and adolescent depressive symptoms. Since previous studies have shown that demographic variables such as gender, age, place of birth, only child or not, parents' education level, and per capita monthly family income were highly correlated with family function and adolescent depressive symptoms (16, 63, 64). In this study, those variables were put in the model as control variables, thus we could exclude the potential influence on them. In the autoregressive cross-lagged model, we also allowed the synchronous correlations among the two latent variables, and the error correlation of the same observed variables at the three measurements. The model fitted data well: χ^2^/df = 4.07, *p* < 0.001, CFI = 0.93, TLI = 0.91, RMSEA = 0.05 (90% CI = [0.046, 0.052]), SRMR = 0.08. Figure 1 showed the standardized path coefficients. To simplify the model, the predictive pathways of the control variables for the family function and adolescent depressive symptoms at the three-time points are not shown in Figure 1.

The results suggested that gender (β = 0.06, *p* < 0.05), father's education level (β = 0.11, *p* < 0.05), and per capita monthly family income (β = 0.08, *p* < 0.05) had a significant predictive effect on T1 family function. The predictive coefficient of gender for T1 and T3 adolescent depressive symptoms was significant (β = 0.08, *p* < 0.05; β = 0.15, *p* < 0.001, respectively). Father's education level had a significant predictive effect on T2 adolescent depressive symptoms (β = −0.09, *p* < 0.05), and only child or not also had a significant predictive effect on T3 adolescent depressive symptoms (β = −0.08, *p* < 0.01). After controlling for the influence of demographic variables, the autoregressive path coefficients of family function were 0.29 and 0.25, respectively; and the autoregressive path coefficients of adolescent depressive symptoms were 0.24 and 0.23, respectively. These results indicated relatively strong autoregressive effects for both family function and adolescent depressive symptoms over 3 years. As for the cross-lagged effects, family function at T1 significantly and negatively predicted adolescent depressive symptoms at T2 (β = −0.09, *p* < 0.01), and adolescent depressive symptoms at T2 significantly and negatively predicted family function at T3 (β = −0.07, *p* < 0.05). However, the prediction of adolescent depressive symptoms at T1 on family function at T2 was not significant, and the prediction of family function at T2 on adolescent depressive symptoms at T3 was also not significant.

## Discussion

From the perspective of family system theory, the current study adopted three waves of longitudinal data with a 3-year time lag to examine the relationship between family function and depressive symptoms among Chinese middle school-aged adolescents. Our study found that both family function and depressive symptoms in adolescents were stable in Grade 7 and Grade 8. However, in Grade 9, there was a significant increase in family function, but a significant decline for adolescent depressive symptoms. The results of the cross-lagged analysis reveal that the associations between family function and adolescent depressive symptoms are dynamic and time-dependent: the family function in Grade 7 negatively influences depressive symptoms of adolescents in Grade 8, after being affected, adolescent depressive symptoms in Grade 8 could also negatively impact subsequent family function in Grade 9. In other words, there is a circular effect between family function and adolescent depressive symptoms.

In our study, we found a slight decrease in family function and a subtle increase in adolescent depressive symptoms between Grade 7 and Grade 8, but neither of them was significant. However, compared to the first 2 years, there were significant changes in family function and adolescent depressive symptoms in Grade 9. In the last year of junior high school, adolescent depressive symptoms significantly declined and their family functioning significantly increased. These findings supported our hypothesis (i). Our results were not consistent with previous studies based on western samples (23, 50), but they were in line with recent researches conducted in China (65, 66). For example, Sun et al. (65) followed 1,419 Chinese junior high school students from thirteen junior middle schools in Beijing for 3 years and found that depressive symptoms in junior high school students was relatively stable from the first to the second year but declined significantly in the third year. Another study, using Chinese rural junior middle school students, also found that family functioning was significantly higher in the third year than in the first and second years (66). Our findings indicate that both family function and adolescent depressive symptoms have their unique developmental characteristics in China.

Regarding the change of family function, possible explanations are that families have to adapt to the children's transition from primary school to secondary school, and they also need to accommodate to students' growing needs for independence and autonomy (48). These may lead to comparatively low family functioning during Grade 7 to Grade 8. At Grade 9, the preparation for senior high school entrance examination in China may enhance autonomy support from parents, promote family harmony, and reduce conflict in the family, thereby making the entire family function more properly. Several reasons for the development trend of adolescent depressive symptoms are as follows. During Grade 7 to Grade 8, students have to adapt to the new peers and new teachers as well as confront the challenges of self-development tasks like self-identity confusion (67), so the depressive symptoms are relatively high. After entering Grade 9, apart from the special attention on entrance examination that makes adolescents regulate their mood to concentrate on learning, the matures of physical and mental development may also contribute to the natural decrease of mild or moderate depressive symptoms in adolescents (68). In addition, our study also showed that family function was significantly and negatively associated with adolescents' depressive symptoms at the same measuring point during three school years. The significantly negative correlation between them also suggests that the two variables share many common changes and have a close linkage.

The results of cross-lagged analysis showed that the impaired family function significantly predicted adolescent depressive symptoms from Grade 7 to Grade 8, which supports our hypothesis (ii). This result is consistent with most previous studies showing that family dysfunction, including more family conflict, lower levels of interactions with parents, and poor family relationships, is a risk factor for adolescents' internalizing problems (28, 37, 69). Early adolescence is a susceptible period for individuals to develop anxiety, depression, and other psychological problems (70). Adolescents who living in an unhealthy family have to cope with various negative life events, which would trigger a series of stress that negatively influencing their cognitive style, leading them slide into depression (71). Meanwhile, a poor family function is also a hazard factor that hampers the development of positive psychological resources (e.g., resilience, cognitive ability, emotional regulation ability) in adolescents (28). These resources are considered as strengths to help adolescents overcome adverse situations and stay away from depression (72, 73). Furthermore, emotional security theory also contends that family instability and interparental conflict could induce the emotional insecurity of children in the family, lead to boosted fear, vigilance and distress, and further contribute to a greater likelihood of emotional problems, including depressive symptoms (74, 75).

Interestingly, our study found no significant effect of family function at Grade 8 on adolescent depression at Grade 9. We speculate that, in early middle school years, family functioning may have a strong effect on adolescent internalizing problems. As adolescents become mature, its influence on adolescents might gradually diminish due to the growing demand for autonomy and independence, as well as the increasing importance of peer relation (76, 77). Furthermore, in the current study, we discovered the opposite direction of predictive effect from adolescent depressive symptoms to family function, supporting our hypothesis (iii). The late appearance of the child effect is in agreement with several longitudinal studies showing that adolescents play a stronger role in the development of their families as children grow older (77). For instance, Georgiou and Fanti (78) found that the relationship between child's behavior problems and mother-child conflict was bidirectional at early ages. But, as time passed by, “child effects” become stronger compared to “parent effects.” Two possible explanations might help us understand the emergence of the child effect. On one hand, in contrast to “upper” pressure from the social environment, adolescent depressive symptoms, as a source of “below” pressure from children's behavior problems, may elicit more intrusive parenting and produce more parenting stress (79, 80). Thus, adolescent depressive symptoms would influence the parent-child relationship and even damaging the communication among family members (48, 49), making the whole family system get into dysfunctional states (48, 49). On the other hand, emotional problems (such as anxiety, depression, etc.) are easily contagious among households' members (45), which may change the whole atmosphere of the family and reduce the cohesion and adaptability of the family. Therefore, during the later middle school years, when the influence of adolescent depressive symptoms on the family has accumulated to a certain degree after a period of time, adolescent depressive symptoms turn to “erode” and “damage” family function.

Although the cross-lagged analysis results indicate that both family function and adolescent depression can serve as a cause and a consequence, the direction of associations between family function and adolescent depression depend on time. That is to say, during the early middle school year, adolescents are easier to get depressed because of impaired family function. But in the later middle school year, adolescents who experienced more depression are more likely to get a decline in their family function. Thus, the results suggest that there is an unexpected circular effect rather than the hypothetical reciprocal effects, thus hypothesis (iv) is not verified. Our findings are not in line with the earlier research finding bidirectional effects between family conflict and adolescent depressive mood (50), as well as the previous research of western adolescents that only discovered a significant effect of childhood behavior problems on family functioning, but not vice versa (23). Our study has unfolded a more comprehensive picture of the dynamic reciprocity between family function and adolescent depressive symptoms, consistent with developmental contextualism emphasizing the interaction between organism and context on development (81). Differences, such as the interval of tracking (i.e., 6 vs. 12 months) and considered covariates (i.e., exclude vs. include of transition) may offer explanations for these inconsistencies between our results and those studies conducted on western culture (23, 50). Of note, in the current study, we found significant predictive effects of family function on adolescent depressive symptoms from Grade 7 to Grade 8, as well as adolescent depressive symptoms on family function from Grade 8 to Grade 9, both with relatively small coefficients (−0.09 and −0.07). Indeed, in longitudinal studies, since the medium predictive effects of a predictor on the outcome are greatly attenuated by the strong stability in the outcome, it is not at all surprising that even the small effect sizes were not trivial, but still meaningful after controlling for the stability effects (82).

Several limitations should be cautiously taken into consideration in this study. First, the results of this study relied on adolescent self-report. This may result in biased outcomes, considering that depressed mood could affect adolescents' perceptions of family conflict and mutual relationships (83). Future studies may take other assessment methods such as observer-rating (e.g., McMaster Clinical Rating Scale) to provide a more objective understanding of family functioning (84). Second, our study only tracked three waves from Grade 7 to Grade 9, thus we may not capture the school transition, such as from primary school to middle school or from middle school to high school, during which great change occurs both for adolescents and the family (85, 86). Future research is necessary to explore the relationship patterns between family function and adolescent depressive symptoms using more data waves including the critical school transition period. Third, the present study has only examined the dynamic interplay between family function and adolescent depressive symptoms. In future studies, it is worthwhile to see if these associations would change due to some socio-demographic factors such as gender (girl vs. boy) or socioeconomic status (economic advantage vs. economic disadvantage). Positive traits of individuals (e.g., resilience) or contextual variables (e.g., peer support) also need to be examined to identify possible buffers between family functioning and adolescent depressive symptoms. Last but not the least, our results were based on a sample of middle school students in China belonging to a collectivistic culture, which may show the influence of cultural differences (54). More data are needed from other cultures to verify the cultural uniqueness of the development trend of family functioning and adolescent depressive symptoms and the interrelationships between them. Additionally, we also need to notice that the auto-regressive cross-lagged models introduce an inherent problem of between- and within-person associations not being disaggregated, which would influence the interpretability of our results.

Despite these limitations, our study contributes to the family-child relations literature by supporting an intertwined developmental relationship of family function and adolescent depressive symptoms. This research also provides an important addition of how such relationships could depend on time, which implying that researchers and practitioners should emphasize different points in different periods when it comes to practice. In the early middle school year, considering the protective impact of family function on adolescent mental health, it is particularly crucial that families should try their best to create an enabling and harmonious environment to prevent their children from mental disorders. While at the late stage of middle school, given the influence of adolescent depressive symptoms on the family system, it is urgently demanded for parents and other family members to understand depression and its effects fully and deeply, thus avoiding excessive erosion of adolescent depressive mood on the whole family. Meanwhile, in view of the interrelated nature of family and adolescents, the role of school and government must not be ignored. For those adolescents living in dysfunctional families, schools should try to reduce the harm caused by adverse family environments via a variety of methods. For instance, schools could educate teenagers on emotional regulation strategies, guide them to adjust negative self-cognition and self-evaluation, cultivate their positive traits. While the government should take supportive policies for healthy family function and promote family education to maximize these adolescents' welfare. For example, the government could encourage or implement the family-based prevention or intervention programs targeting both children and their families at risk, which may contribute to the virtuous circle between family and adolescents and produce long-last beneficial effects.

## Conclusion

In conclusion, the present study found that both family function and depressive symptoms underwent a certain change during the three junior high school years. But the negative cross-sectional correlation between family function and adolescent depressive symptoms remained significant and stable across three academic years. Moreover, during the early middle school year, poor family function significantly affected subsequent adolescent depressive symptoms. While in the later middle school year, adolescent depressive symptoms significantly influenced subsequent family function. Given the intertwined nature of the family function and adolescent depressive symptoms, family-based intervention should be a promising method both for adolescents and their families.

## Data Availability Statement

The original contributions presented in the study are included in the article/supplementary material, further inquiries can be directed to the corresponding author/s.

## Ethics Statement

The studies involving human participants were reviewed and approved by the Human Research Ethics Committee of Shen Zheng University. Written informed consent to participate in this study was provided by the participants' legal guardian/next of kin.

## Author Contributions

EW and JZ designed the study and directed its implementation, did the literature search, and wrote the manuscript. EW, SP, and BZ reviewed the manuscript and revised it critically. All authors contributed to and have approved the final manuscript.

## Funding

The study was sponsored by National Social Science Foundation of China (Grant Number: 16CSH049); Shenzhen Basic Research Grant (Grant Number: 2019SHIBS0003); Guangdong Basic and Applied Basic Research Foundation (Grant Number: 2021A1515011330); and the Guangdong Education and Science Project of the 13th Five-Year Plan (Grant Number: 2018GXJK238).

## Conflict of Interest

The authors declare that the research was conducted in the absence of any commercial or financial relationships that could be construed as a potential conflict of interest.

## Publisher's Note

All claims expressed in this article are solely those of the authors and do not necessarily represent those of their affiliated organizations, or those of the publisher, the editors and the reviewers. Any product that may be evaluated in this article, or claim that may be made by its manufacturer, is not guaranteed or endorsed by the publisher.
